# Popliteal Aneurism Successfully Treated by Compression

**Published:** 1848-06

**Authors:** 


### ECLECTIC DEPARTMENT,

### AND SPIRIT OF THE MEDICAL PERIODICAL PRESS

---

[Communicated for the Boston Medical and Surgical Journal.]

*Popliteal Aneurism successfully treated by compression.* By J. Knight, M. D. and D. A. Tyler, M. D.

On the 18th of October, 1847, I was consulted, at the request of Dr. D. A. Tyler, of this city, by Henry Johnson, a wood-sawyer about 48 years old, for a tumor in the left ham—the left leg being the limb he uniformly stood upon whilst at his business. The tumor Dr. Tyler considered to be an aneurism of the popliteal artery. For several months he had suffered from pain in the limb, especially below the knee, which the patient thought was rheumatism; and about two months before, had discovered a small tumor in the ham, which pulsated from the beginning, and which, as well as the pain, had been gradutally increasing. I found the tumor of such a size as to occupy the whole of the popliteal space, and to press strongly upon the tendons of the flexor muscles, particularly those upon the outside of the limb. The leg, below the knee, was very painful and largely œdematous. All the symptoms of aneurism were so strongly marked as to leave no doubt concerning the the nature of the affection. The action of the artery and the pulsation of the tumor were easily suppressed by very moderate pressure upon the femoral artery, either where it passes over the bone of the pubis or in the groin, or still lower down where it passes under the sartorius muscle. The patient was directed to lie in bed for a few days; and to relieve the severe pain he suffered, laudanum was administered in doses of 30 or 40 drops, and, when necessary, some of the common cathartics. By these means, employed for a week, the œdema of the leg was cured, and the pain very much diminished: indeed, while in bed he was free from pain. The tumor, however, was unchanged in site or character.

We now concluded to attempt a cure by pressure on the femoral artery above the tumor, upon the plan recently practiced by Hotton, Bellingham and others. For this purpose we employed successively all the means which have been described—the hoop tourniquet, the calliper-shaped instrument, the common tourniquet, guarding the limb against the pressure of the strap by encasing it in thick sole leather, and by a variety of other mechanical contrivances. There was no difficulty in controlling the artery, either diminishing its action or suppressing it, by any of the instruments employed. By whatever instruments, however, the pressure was made, however carefully it was guarded, whether continued upon one point only, or shifted from one part of the artery to another, the pain in a short time became so severe that it could not be borne. There was no difference in this respect whether the limb was left uncovered or enveloped in a bandage from the toes upward. The pain was not in the part pressed upon by the instruments, but in the whole limb below, and was felt equally in the thigh, especially ,the outside of it, and the leg below the knee. The pain usually began in 25 or 30 minutes after the pressure was applied, and became intolerable in 15 or 20 minutes longer, so that it could not be continued in any instance longer than an hour. These efforts were continued at intervals for 8 or 10 days and, as nothing had been gained by them, were abandoned. Others have met with the same difficulty, and have been compelled to apply a ligature to the artery.

Before resorting to this, I concluded, with the concurrence of Dr. Tyler, to try manual pressure upon the artery. To accomplish this, a sufficient number of assistants was procured from the members of the medical class, who cheerfully offered their services. They were divided into relays—two keeping up the pressure for four or five hours, relieving each other every half hour, and these succeeded by two others, and so on, Sufficient pressure to suppress the pulsations of the tumor was found to be most easily made with the thumb or fingers, without a compress, upon the artery, as it passes over the os pubis; and the directions given to the assistants was to keep up this amount of pressure as nearly continuous as possible. This treatment was commenced at 3, P. M. No pain of consequence was produced by it for five or six hours; and then it was not severe, so that the patient was quieted by one-eighth of a grain of morphine once or twice repeated, and slept for the most part of the night. About eight hours after the pressure was applied, the temperature of the limb was diminished, and it appeared shrunken in size. At 11 o'clock the next morning, 20 hours from the commencement of the treatment, upon removing pressure from the artery, the tumor had diminished very little, if at all, and pulsated about as strongly as ever, but the tibial arteries could not be felt. Probably the blood had ceased to enter them from the tumor during the night, in consequence of a coagulum forming in the artery where it left the aneurism. Upon examining the parts the next morning, about 40 hours from the commencement of the treatment, the tumor was found nearly one-third smaller in size, firm and unyielding on pressure, and entirely without pulsation. All treatment was discontinued. The femoral artery pulsated with its usual strength in the groin, and distinctly as far as its passage through the tendon of the adductor muscles. Between this point and the tumor, it could not be felt. Several of the anastomosing arteries, especially one upon the inside of the limb, could be felt pulsating strongly and enlarged in size.

From that time to this, a period of five months, no change has taken place in the limb, except that the tumor has gradually diminished in size so as now scarcely to be felt; and the leg, which was at first cold and weak, has nearly regained its natural temperature and strength.

The peculiarity of this case consists in the manner of making the pressure upon the artery, by the hands of assistants instead of mechanical contrivances; and in the slight pain the patient experienced from it. The advantages of this mode of making pressure are such, that I should at once resort to it, if, upon trial, pressure by instruments was found to occasion much pain. It is interesting to look back upon the improvement which has been made in the treatment of aneurism during the past one hundred years. It is but seventy years since the only treatment ordinarily adopted for an external aneurism, consisted in cutting into it, emptying it of its blood, and applying a ligature to the two ends of the artery. The danger of this operation, from inflammation and from secondary hæmorrhage, was such that a large portion of the patients died.

The first and greatest improvement, and that which has led to all the others, was the plan devised, not guessed at, but fairly reasoned out with great sagacity from well-known physiological and pathological laws, by Mr. Hunter, of placing a ligature upon the artery at a distance from the aneurismal tumor. The success of this mode of operating has been so great, that surgeons had almost ceased to look for any improvement on it.

Upon this plan, however, the treatment by pressure is a great improvement. It is so simple, so easily practiced even by those not familiar with surgical operations, and as far as it has been tried, so entirely free from danger, that it well deserves to be employed in all cases to which it is appropriate. The principal objection to this plan, is the pain which it sometimes produces. This is mentioned in the cases which have been stated, as often severe, and, in some, intolerable. This objection is removed, if it should be found, that in other cases, as in the one which I have stated, manual, in the place of instrumental pressure, is so easily borne. One case does not prove that it will be so, but it is sufficent to encourage others to make trial of it. In some cases of aneurism, which are so near to the trunk of the body that pressure cannot be made upon the artery between it and the heart, it may perhaps be made upon the distal side of the tumor with success. If it is so situated that efficient pressure can be made upon the artery beyond the tumor, and before any considerable branch is given off from it, coagulation of the blood will probably take place, and a cure be effected.

The treatment by pressure would seem, also, to be adapted to some cases of traumatic hæmorrhage; those especially where a deeply-seated artery is divided at the bottom of a narrow wound, as in the palm of the hand or the sole of the foot. The trouble and anxiety which attend such cases, are familiar to every surgeon. In such cases, continuous pressure for a long time would probably not be necessary. It is a well-known fact that arteries, when wounded, bleed in paroxysms at varying intervals, usually of several hours, and that obvious symptoms precede the hæmorrhage. These are, increased heat, pain, and throbbing of the arteries of the part wounded. If, when these symptoms appear, pressure is made upon the arteries above, as as upon the brachial artery, when the wounded artery is in the hand, the period of increased action of the vessels of the part and of hæmorrhage may pass by, when the pressure can be removed, to be renewed again with the re-appearance of the symptoms, and continued thus at intervals until the wound in the artery is healed. I make this suggestion for those into whose hands a case of this kind may fall. The trouble to the surgeon, which attends this mode of treatment, is not to be taken into the account when the life of the patient is involved, or when he is to be saved from a severe surgical operation.

New Haven, April 25, 1848.

